# STATIN USE REDUCES ALL-CAUSE MORTALITY IN NURSING HOME RESIDENTS WITH DEMENTIA

**DOI:** 10.1093/geroni/igad104.3745

**Published:** 2023-12-21

**Authors:** Paul Gellert, Julie L O’Sullivan, Raphael Kohl, Sonia Lech, Laura Romanescu, Johanna Schuster, Adelheid Kuhlmey, Sevil Yasar

**Affiliations:** Charité, Charité, Berlin, Germany; Charité, Berlin, Berlin, Germany; Charité, Berlin, Berlin, Germany; Charité Universitätsmedizin Berlin, Berlin, Berlin, Germany; Charité, Berlin, Berlin, Germany; Charité, Berlin, Berlin, Germany; Charité-Universitätsmedizin Berlin, Berlin, Berlin, Germany; Johns Hopkins University School of Medicine, Baltimore, Maryland, United States

## Abstract

**Background:**

Statins are recommended for the prevention of primary and secondary cardio and cerebrovascular events. However, little is known about the benefits of statin therapy in older adults with dementia. Therefore, this retrospective cohort study aims to investigate the association between statin use and all-cause mortality using health insurance claims data of nursing home residents with and also without dementia.

**Methods:**

Time-variant Cox proportional hazards models were used to evaluate the association of statin use with all-cause mortality and adjusted for potential confounders.

**Results:**

Overall, N=326,669 individuals were included in the analysis (mean [SD] age 84.02 [8.45] years; 74% women). During a mean observation period of 2.42 years (SD=1.59), a total of 239,321 deaths occurred. The fully adjusted models showed that participants with dementia statin users compared to non-users had a 20% lower risk for all-cause mortality (HR 1.20; 95%CI: 1.19–1.22, p<.001). In a subanalysis, similar trends remained within those with history of atherosclerotic cardiovascular disease (ASCVD) (HR 1.22; 95%CI: 1.21–1.24, p<.001) and without ASCVD disease (HR 1.12; 95%CI: 1.09–1.16, p<.001); and when stratifying by dementia type, Vascular dementia (VaD) (HR 1.27; 95%CI: 1.22-1.32, p< 0.001) and Alzheimer’s disease (AD) (HR 1.19; 95%CI: 1.13-1.26, p< 0.001). Trends remained similar when stratifying by statin intensity, age, nursing home care level required, and sex.

**Conclusion:**

Our study demonstrates the benefits of statin therapy in highly vulnerable nursing home residents with dementia. This contributes to filling the knowledge gap regarding the harms and benefits of statin therapy in older adults.

